# Heterozygous TREM2 (p.W44X) and PSEN1 (p.A431T) mutations in two Peruvian families with familial Alzheimer’s disease: expanding the genetic landscape in underrepresented populations

**DOI:** 10.3389/fnins.2025.1724380

**Published:** 2026-02-02

**Authors:** Claudio Villegas-Llerena, Solange R. Paredes-Moscosso, María Luisa Guevara-Fujita, Daisy Obispo, Nilton Custodio, Rosa Montesinos, José F. Parodi, Oscar Flores-Flores, John Hardy, Ricardo Fujita

**Affiliations:** 1 Centro de Investigación de Genética y Biología Molecular (CIGBM), Instituto de Investigación, Facultad de Medicina Humana, Universidad de San Martin de Porres, Lima, Peru; 2 Research Unit, Instituto Peruano de Neurociencias, Lima, Peru; 3 Universidad de San Martín de Porres, Facultad de Medicina, Centro de Investigación del Envejecimiento, Lima, Peru; 4 Department of Neurodegenerative Disease, UCL Queen Square Institute of Neurology, University College London, London, United Kingdom; 5 UK Dementia Research Institute, London, United Kingdom

**Keywords:** Alzheimer’s disease, genetics, Latin America, mutation, PSEN1, TREM2

## Abstract

Alzheimer’s disease (AD) accounts for up to 70% of all dementia cases, affecting an estimated 23–35 million people worldwide. According to the World Health Organization (WHO), the number of AD cases in Latin America, including Peru, is expected to quadruple by 2050. However, these populations remain underrepresented in research, diagnostics, and care. Early-onset Alzheimer’s disease (EOAD), characterized by symptom onset before the age of 65, has been shown to have a strong genetic component, making it valuable for genetic studies. Identifying EOAD-associated mutations in underrepresented populations is crucial for uncovering pathogenic variants that may provide new insights into the disease’s mechanisms. In this article, we present two Peruvian families with early and late onset AD in whom whole-exome sequencing (WES) revealed heterozygous variants associated with AD. In family AD002, we found a heterozygous variant in *TREM2* (c.132G > A; p.W44X), a protein-truncating mutation. The proband and 17 family members participated in genetic testing, of which 04 members were carriers of the mutation. This is the first *TREM2*-associated mutation reported in the Peruvian population. In family AD009, a novel heterozygous variant in *PSEN1* (c.1291G > A; p.A431T) is reported. The proband and 11 family members participated in genetic testing, of which 05 were carriers of the mutation (02 affected siblings and 03 unaffected relatives). This is the first report of *PSEN1* A431T associated with AD. Overall, our findings suggest that TREM2 p.W44X is a likely-pathogenic variant while PSEN1 p.A431T is a candidate variant of uncertain significance (VUS) associated with AD; both genetic variants warrant further investigation.

## 1 Introduction

Genetic studies have provided crucial insight into Alzheimer’s disease (AD) pathogenesis. Most cases of AD have no clear mode of inheritance and occur after the age of 65 years: Late-Onset AD (LOAD). Early studies using genetic linkage approaches associated chromosome 21 with Early-Onset Familial AD (EOFAD) cases (St George-Hyslop et al., 1987; Tanzi et al., 1987). These findings paved the way for the discovery of pathogenic mutations in amyloid precursor protein (APP; MIM: 104760; Goate et al., 1991), presenilin 1 (PSEN1; MIM: 104311; Sherrington et al., 1995) and PSEN2 (MIM: 600759; Levy-Lahad et al., 1995)—all of which participate in Amyloid beta processing. At around the same period, researchers identified the apolipoprotein E4 (APOE4; MIM 107741) variant, a key susceptibility allele for late-onset Alzheimer’s disease (Strittmatter et al., 1993). Furthermore, the advent of genome-wide association studies (GWAS) has allowed the identification of more than 80 loci associated with AD (Andrews et al., 2023; Harold et al., 2009; Hollingworth et al., 2011; Lambert et al., 2009). To date, most genetic studies on AD have predominantly included populations of European ancestry; with the concomitant underrepresentation of non-European populations, potentially leading to reduced diversity in AD genetic research (Miyashita et al., 2023).

Next,-generation sequencing (NGS) has allowed the discovery of rare variants associated with increased AD risk. Notably, the combined use of whole-exome sequencing (WES) and GWAS led to the identification of rare heterozygous *TREM2* variant (Guerreiro R. et al., 2013; Jonsson et al., 2013), which confer a risk similar to a single APOE4 copy (a single APOE ε4 allele increases risk of developing LOAD by ∼3–4-fold; Long and Holtzman, 2019). Unlike most GWAS-identified SNPs, AD-associated *TREM2* variants are primarily coding mutations, facilitating *in vitro* and *in vivo* modeling. *TREM2* has also been implicated in other neurodegenerative diseases, reinforcing its key role in shared pathogenic mechanisms and highlighting the innate immune system’s connection to neurodegeneration (Jay et al., 2017). This last connection was made because *TREM2* is exclusively expressed on immune cells (mainly microglia in the brain), which provides evidence that immune dysregulation can be a primary, causal contributor to neurodegeneration (Ransohoff, 2016). Furthermore, earlier functional work has demonstrated TREM2 role in phagocytosis and immune signaling (Colonna and Wang, 2016). Additionally, recessive loss-of-function mutations in *TREM2* had been previously shown to cause Nasu–Hakola disease (Klünemann et al., 2005; Paloneva et al., 2002), linking the gene to broader neurodegenerative phenotypes.

Remarkably, familial AD is associated with the genes that encode PSEN1, PSEN2, and APP, all of which participate in the normal processing of the Aβ peptide (Cacace et al., 2016), giving support to the “amyloid hypothesis.” Interestingly, *PSEN1* variants have been identified in up to 70% of cases of familial AD, with more than 220 pathogenic mutations identified so far. *PSEN1* mutations lead to the most severe forms of AD, exhibiting full penetrance and an onset as early as 25 years of age (Andrade-Guerrero et al., 2023).

Importantly, the majority of AD genetic studies have been performed in European-ancestry populations, resulting in limited representation of Latin American, African and other ethnic groups. Recent reports suggest that population-specific variants and ancestry-related haplotypes may influence both AD risk and phenotype (Andrews et al., 2023; Marca-Ysabel et al., 2021), highlighting the need for genetic studies in diverse populations. In a recent report from the ReDLat consortium, focusing on AD and FTD in admixed Latin American participants, with a particular emphasis on families, they identified a total of 17 pathogenic variants, a pathogenic C9orf72 expansion, and 44 variants of uncertain significance (VUS). In families with AD, the authors identified a novel variant in the PSEN1 gene, c.519G > T (p.L173F), as well as other previously described variants. As our own study, this is one of the first snapshots of genetic contributors to AD (and other dementias) in family cohorts from Latin America (Acosta-Uribe et al., 2024).

In line with the ReDLat consortium report, we aimed to expand the genetic characterisation of Alzheimer’s disease in underrepresented Latin American populations by evaluating 14 Peruvian families with early- and late-onset forms of AD diagnosis using whole-exome sequencing. We identified a heterozygous protein-truncating variant in *TREM2* (c.132G > A; p.W44X) in family AD002 and a novel heterozygous *PSEN1* variant (c.1291G > A; p.A431T) in family AD009. Segregation analyses revealed that four members of family AD002 carried the *TREM2* variant, whereas five relatives in family AD009 carried the *PSEN1* substitution. Based on genetic, clinical, and segregation evidence, our findings support TREM2 p.W44X as a likely pathogenic variant in this population and indicate that PSEN1 p.A431T represents a candidate variant of uncertain significance with potential relevance to familial and early-onset AD. Together, these results highlight the importance of incorporating genetically diverse populations into AD research and underscore the need for further functional and population-based studies to clarify the pathogenicity of these variants.

## 2 Materials and methods

### 2.1 Individuals

A total of 14 Peruvian families were evaluated at two different centers: (1) Instituto Peruano de Neurociencias (IPN) and (2) Centro de Investigación del Envejecimiento (CIEN), both located in Lima, Peru.

**Figure 1:**
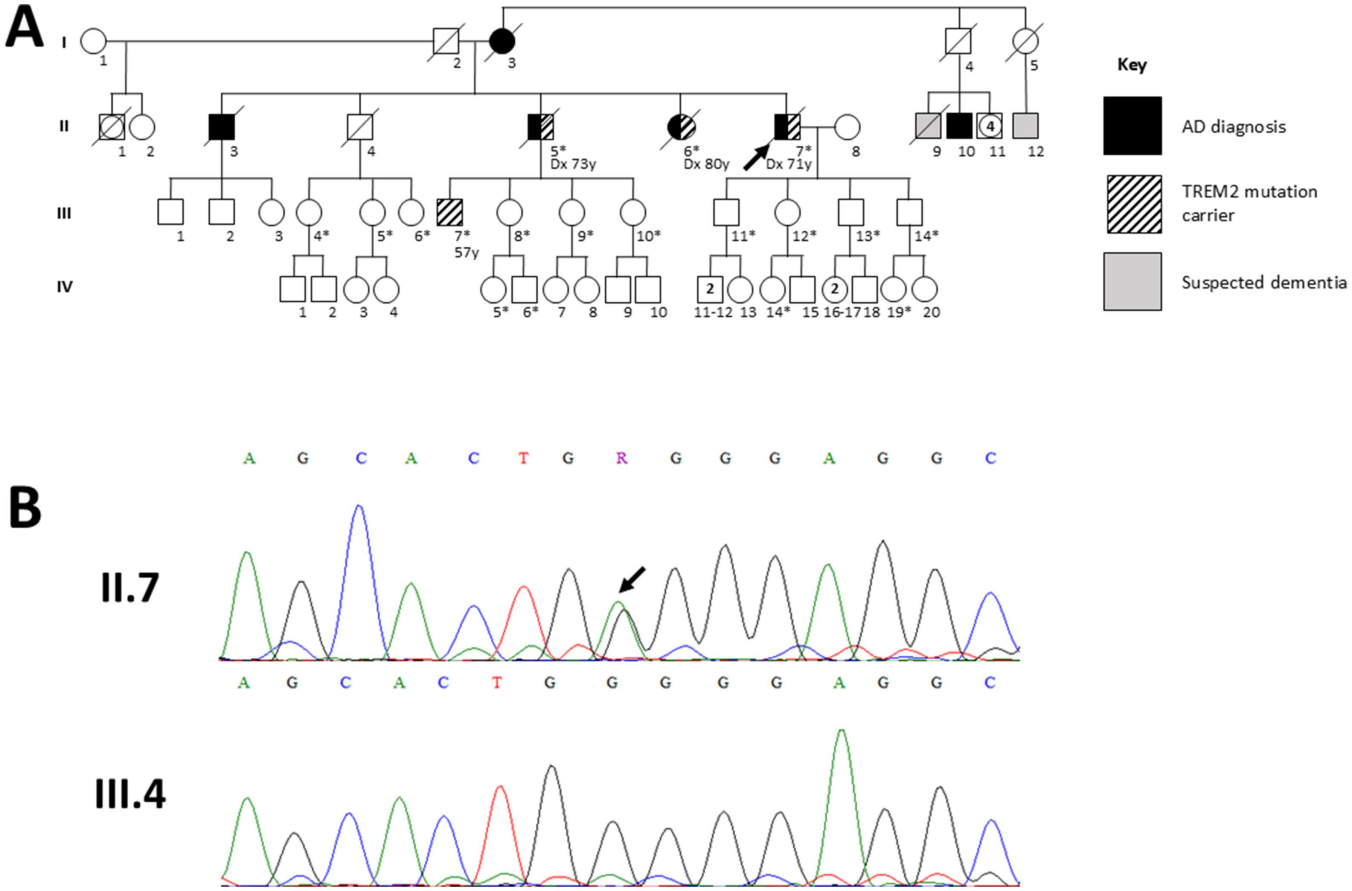
Pedigree of family AD002 and Sanger sequencing results for the TREM2 p.W44X variant. **(A)** Pedigree showing individuals from family AD002 evaluated in this study. Squares and circles represent male and female participants, respectively; filled symbols indicate individuals clinically diagnosed with Alzheimer’s disease, and unfilled symbols represent unaffected individuals. Hatched symbols denote heterozygous carriers of the TREM2 p.W44X (c.132G > A) variant, confirmed by Sanger sequencing. Asterisks (*) indicate family members who consented to genetic testing. The proband (II.7, indicated by an arrowhead) was diagnosed at age 71 (indicated in the figure as Dx); affected siblings II.5 and II.6 were diagnosed at 73 and 80, respectively. III.7 is an asymptomatic carrier (evaluated at 57, which is also his current age), with normal neuropsychological performance. The inheritance pattern for this variant remains inconclusive. **(B)** Representative Sanger sequencing chromatograms showing the heterozygous G > A substitution at codon 44 (TGG → TGA) in the proband (II.7), compared with the wild-type sequence in an unaffected non-carrier (III.4). The exact position of the variant is indicated by an arrowhead.

Family AD002’s proband (Figure 1; Individual II.7) and other 17 family members participated in the genetic testing. Only the proband and individuals II.5, II.6 and III.7 were clinically evaluated at IPN.

**Figure 2:**
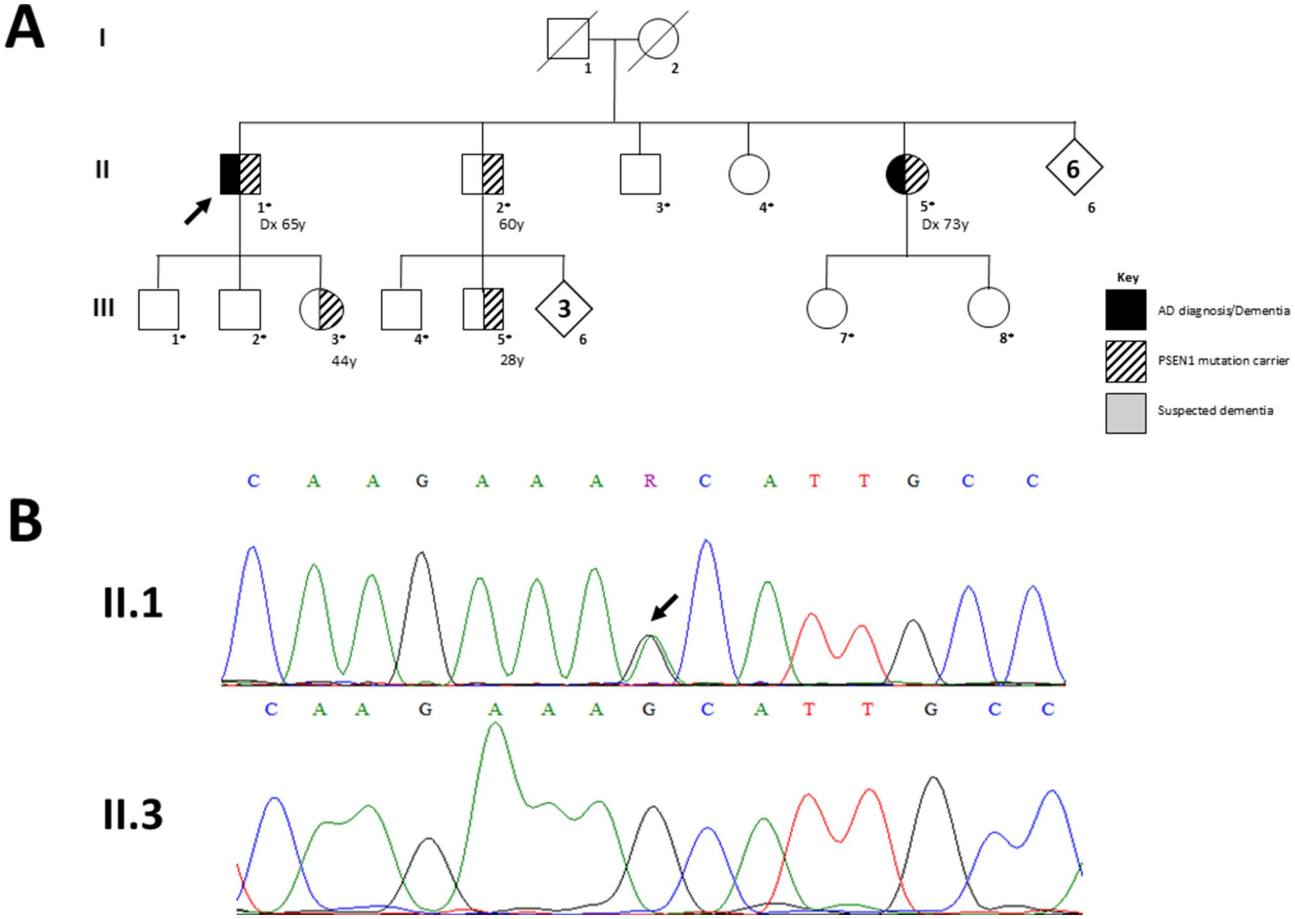
Pedigree of family AD009 and Sanger sequencing results for the *PSEN1* p.A431T variant. **(A)** Pedigree of family AD009. Squares and circles represent male and female participants, respectively; filled symbols indicate individuals clinically diagnosed with Alzheimer’s disease, while unfilled symbols represent unaffected individuals. Hatched symbols denote heterozygous carriers of the *PSEN1* p.A431T (c.1291G > A) variant. Family members who participated in genetic testing are marked with asterisks (*). The proband (II.1, arrowhead) was diagnosed at 65 years, while his affected sibling II.5 was diagnosed at 73 years (indicated in the figure as Dx). Three additional relatives (II.2, III.3, and III.5) were mutation carriers but remained clinically unaffected at ages 60, 44, and 28, at the time of publication (indicated in the figure). The inheritance pattern for this variant remains inconclusive. **(B)** Sanger sequencing chromatograms showing the heterozygous G > A substitution in the proband (II.1) compared with the wild-type sequence from his non-carrier brother (II.3). The exact position of the genetic variant, corresponding amino acid change (Ala→Thr at position 431), is indicated by an arrowhead.

Family AD009’s proband (Figure 2; Individual II.1) and other 11 family members participated in the genetic testing. Only the proband was clinically evaluated at CIEN. Individual II.5 was clinically evaluated and diagnosed in the United States.

All volunteers gave their informed consent to participate. This investigation received ethical approval from the “Comité de Ética en Investigación de la Universidad de San Martín de Porres” (IRB IORB00003251 OHRP/FDA).

### 2.2 Clinical characterization

Participants underwent a structured family history interview, with information obtained from both patients and their study partners. A family history was considered positive if at least two first- or second-degree relatives had been diagnosed with dementia or another neurodegenerative disorder. Medical history and comprehensive neuropsychological assessments were conducted as described in the results section. Cerebrospinal fluid and imaging biomarkers were not performed as, to the best of our knowledge, there is currently no availability of this kind of studies in a clinical setting within Peru. Similarly, blood-based biomarkers such as plasma Aβ, phosphorylated tau (p-tau), GFAP, and neurofilament light (NfL) were not available at the time of clinical evaluation and were not included in the diagnostic criteria.

### 2.3 Genetic testing

Genomic DNA was isolated from ethylenediamine tetraacetic acid (EDTA)-treated whole blood samples (~3 mL) using the salting-out procedure with minor modifications (Miller et al., 1988). The samples were subsequently quantified and checked for purity using a NanoDrop spectrophotometer (Thermo Scientific, Waltham, MA, United States). Purified DNA was sent to Macrogen (Korea) for library construction and Whole-Exome sequencing (WES). DNA libraries were constructed using the SureSelect XT Human All Exon V6–bait library: S07604514 (Agilent Technologies, Santa Clara, CA, United States), and WES was performed using 150 bp paired-end reads and sequenced on a NovaSeq 6,000 platform (Illumina, United States) according to the manufacturer’s instructions.

The raw exome data (fastq files) received from Macrogen was then analyzed *in-house* at our laboratory. Downstream variant analysis was restricted to a curated panel of 119 genes with established or suspected involvement in neurodegenerative diseases (Supplementary Table 1), including Alzheimer’s disease, frontotemporal dementia, Lewy body disease, prion diseases, and hereditary leukoencephalopathies. These genes were selected based on prior literature, inclusion in clinically validated diagnostic panels, known biological relevance to amyloid, tau, lipid metabolism, or microglial pathways, and their presence in the OMIM and ClinVar databases as harboring pathogenic or likely pathogenic variants associated with cognitive impairment. This targeted approach was chosen to prioritize variants with the highest prior probability of clinical relevance while avoiding false-positive findings in a small cohort. Reads were aligned to the GRCh37/hg19 human reference genome with BWA, and variant calling and annotation was performed using VarScan, Variant Effect Predictor (VEP), and SnpEff/Sift tools. The selected variants were evaluated with *in silico* predictors for potential deleterious effects on protein function and pathogenicity (i.e., SIFT, PolyPhen-2, MutationTaster2, Human Splice Finder).

Once a variant of interest (VOI) was identified through WES in a proband, their remaining family members were screened only for the VOI by polymerase chain reaction (PCR) amplification and Sanger sequencing in-house. Genotyping was performed using specific PCR primers: *TREM2* exon 2 region was amplified using forward primer 5′-TCCTTCAGGGCAGGATTTTT-3′ and reverse primer 5′-AGTGGGTGGTTCTGCACAC-3′; *PSEN1* exon 12 region was amplified using forward primer 5’-CTTCCAGATTGAATGAACGTCTG-3′ and reverse primer 5′- CCGGGAATCTTGACTTTGTTAG-3′; while *APOE* (rs7412 and rs429358) was amplified using forward primer 5′-AGCCCTTCTCCCCGCCTCCCACTGT-3′ and reverse primer 5′-CTCCGCCACCTGCTCCTTCACCTCG-3′. Sanger sequencing was performed using the purified PCR products and BigDye Terminator v3.1 Cycle Sequencing Kit (Thermo Fisher Scientific, United States). The sequencing reactions -forward and reverse- were run on a 3,500 ABI Genetic analyzer (Applied Biosystems Inc., United States) and the sequence data was analyzed by BioEdit Sequence Alignment Editor version 5.0.9 (Hall, 1999). Determination of the *APOE* status was only performed in probands, first inferred from WES sequencing and later confirmed by Sanger sequencing.

## 3 Results

A total of 14 probands from 14 AD affected families were recruited initially, all of which went through WES analysis. Of these, we only identified AD-related mutations in 02 probands and their relatives. These two familial cases are reported as follows:

### 3.1 Family AD002

This family was evaluated and treated at IPN since 2017. The pedigree and representative image of the Sanger sequencing confirmation of the mutation are shown in Figure 1.

### 3.2 Index patient clinical description

Proband II.7 was referred to the IPN by his family, who noticed changes on his behavior at least 6 years before their consultation (probable age of onset 65yo). No previous medical record was provided, no other relevant medical history was reported by family members, including substance misuse. The proband was evaluated at 71 years old, with a Mini-Mental Status Examination (MMSE) score of 29/30 (Normal cognition), Clinical Dementia Rating (CDR) score of 0.5 (Very mild impairment), Pfeffer Functional Activities Questionnaire (FAQ) score of 1/30 (Normal/No functional impairment), ADAS-Cog 13 score of 20/85 (Mild-to-moderate Alzheimer’s disease), Alzheimer’s Disease Cooperative Study–Activities of Daily Living (ADCS-ADL) score of 75, Clock Drawing Test (CDT) score of 8/10 (normal aging or early impairment). Based on the results of clinical evaluation, a diagnosis of dementia of Alzheimer’s type was made. ApoE status was ε3/ε3. No imaging was available. Proband died at 77 years of age.

### 3.3 Clinical description of other family members

#### 3.3.1 II.5—AD016

Family member II.5 was referred to the IPN by his family almost at the same time as his brother. No previous medical record was provided, no other relevant medical history was reported by family members, including substance misuse. The family reported that the mother and one older sibling of the proband were diagnosed with dementia at 75yo and 70yo, respectively. II.5 was evaluated at 73 years old, with an MMSE score of 23/30 (mild cognitive impairment), CDR score of 1 (Mild dementia), FAQ score of 7/30 (mild functional impairment), ADAS-Cog 13 score of 31/85 (mild-to-moderate Alzheimer’s disease), ADCS-ADL score of 71, CDT score of 7/10 (normal aging or early impairment). Thus, he was also diagnosed with dementia of Alzheimer’s type. No cerebral imaging was available. Patient II.5 died at age 77.

#### 3.3.2 II.6—AD027

II.6 was evaluated at a private clinic in Lima, Peru. She was diagnosed with AD at 80yo. No medical record was provided, no other relevant medical history. The participant died at age 83.

#### 3.3.3 III.7—AD022

III.7 is the nephew of the proband and was referred to IPN at age 57, after being found to be a carrier of the heterozygous *TREM2* W44X mutation. Clinical evaluation was as follows, MMSE score of 29/30 (Normal cognition), CDR score of 0 (No impairment), FAQ score of 0/30 (Normal / No functional impairment), ADAS-Cog 13 score of 8/85 (Normal cognition / very mild symptoms), ADCS-ADL score of 78, CDT score of 10/10 (normal). Currently, he is not diagnosed with dementia of any type.

### 3.4 Family AD009

Family AD009 was evaluated and treated at CIEN since 2018. The pedigree and a representative image of the Sanger sequencing confirming the heterozygous mutation are shown in Figure 2.

### 3.5 Index patient clinical description

Proband II.1 was referred to CIEN by his family. No previous medical record was provided, no other relevant medical history was reported by family members, including substance misuse. The proband was evaluated at 65 years of age, and presented anomic aphasia. The clinical diagnosis was determined by physicians at CIEN using standard diagnostic criteria. Based on the results of clinical evaluation, a diagnosis of dementia of Alzheimer’s type was made. ApoE status was ε3/ε3. Proband is currently 72yo, he is still able to move independently but is showing signs of rigidity/spasticity. He is currently taking memantine.

### 3.6 Clinical description of other family members

#### 3.6.1 II.2, III.3 and III.5

In these cases, unaffected carriers were not invited for clinical evaluation. Current ages are 60yo, 44yo and 28yo, respectively.

#### 3.6.2 II.5

This participant was clinically diagnosed with dementia in the US at 73yo. Daughter refers that her mother showed mild behavior changes at age 60.

## 4 Discussion

In this study, two AD-related mutations in two different Peruvian families are reported. The first reported mutation in this study is a heterozygous variant in *TREM2* (TREM2 W44X; rs104894001; Chr6:41161522 G > A). This mutation was found in the proband (II.7), two affected siblings (II.5, II.6) and one asymptomatic nephew (III.7), all of whom were clinically evaluated at IPN.

*TREM2* gene encodes for the transmembrane receptor, Triggering Receptor Expressed on Myeloid Cells 2, which has been shown to modulate microglial survival, activation, phagocytosis, maintenance of brain homeostasis and the inflammatory response to injury or neurodegeneration (Zgorzynska, 2024). The reported mutation is in the second exon of the gene (out of five) and consist in a transition G > A, which introduces a premature stop codon in place of tryptophan 44 in the TREM2 protein. This variant was previously reported in homozygosity in a Bolivian patient affected by Nasu-Hakola disease (NHD), also known as polycystic lipomembranous osteodysplasia with sclerosing leukoencephalopathy (PLOSL), a rare autosomal-recessive disease characterized by bone cysts and early onset frontotemporal dementia (FTD; Paloneva et al., 2002). Interestingly, different studies have shown heterozygous rare variants in *TREM2*, including those that cause NHD/PLOPSL in homozygous state, predispose individuals to AD, suggesting that the reduced TREM2 function is key to the pathogenesis seen in AD (Guerreiro R. et al., 2013).

The TREM2 p.W44X mutation is one of six reported mutations within exon 2that cause premature truncation of the protein product (E14X, Q33X, W44X, W78X, W198X and W191X). Of these, the closest truncating mutation, TREM2 Q33X, has been found in at least six rare cases of NHD/PLOSL in homozygosity: two Italian siblings (Soragna, 2003), two Belgian siblings (Klünemann et al., 2005), a Turkish case (Guerreiro R. J. et al., 2013) and another Italian case (Ghezzi et al., 2017). The latter showed signs of amyloid deposition, detected by florbetapir-PET imaging at age 39. Interestingly, both parents of the proband were heterozygous carriers of the Q33X variant and were cognitively normal at age 72 but also showed signs of Aβ deposition. The Q33X mutation has been widely described in homozygosity to cause NHD/PLOSL, while heterozygous carriers have been shown to have increased susceptibility to FTD and AD (Borroni et al., 2014; Song et al., 2017), further suggesting a similar effect of TREM2 W44X. Nevertheless, several other studies failed to confirm the association between Q33X and AD (Benitez et al., 2013; Cuyvers et al., 2014; Guerreiro R. et al., 2013; Jin et al., 2014; Pottier et al., 2013).

Throughout the years, several genetic studies have identified more than 60 different mutations in *TREM2* that are linked to an increased risk of different neurological diseases (Zgorzynska, 2024). Of particular interest are those linked to late-onset AD (LOAD), with R47H (rs75932628) being the most extensively studied. First identified in 2013 by two independent groups (Guerreiro R. et al., 2013; Jonsson et al., 2013), this rare variant elevates AD risk by ~2–4 fold, comparable to APOEε4. The *TREM2* R47H mutation exacerbates neuroinflammation and promotes Aβ protofibril formation, further contributing to AD pathology. This mutation has been associated with the disease in European and Afro-American populations, but not in Asians, which highlights the importance of carrying genetic studies in different populations. Other *TREM2* variants suggested as risk factors for developing LOAD include mutations in exon 2 (Y38C, R62H, D87N, T96K), exon 3 (H157Y) and exon 4 (L211P; Durmanova et al., 2022).

In our reported family, the described mutation is found in heterozygosity in four carriers (three affected and one non-affected, see Figure 1). This variant is predicted to result in a truncated protein lacking the transmembrane and cytoplasmic domains. The truncated polypeptide may retain only a small portion of the Ig-like V-type domain (amino acids 29 to 44), and the complete signal peptide (amino acids 1 to 18). Remarkably, in a patient homozygous for the truncating TREM2 Q33X mutation (Kleinberger et al., 2014), reported very low but detectable levels of sTREM2 in plasma and CSF, as measured by ELISA, indicating that even severely truncated TREM2 can still give rise to shed extracellular fragments. By analogy, it is conceivable that W44X might also allow production of residual sTREM2; however, we did not measure plasma or CSF sTREM2 in our carriers, and no studies to date have directly quantified sTREM2 in individuals with the W44X variant. Thus, any inference about sTREM2 production in W44X carriers remains speculative and should be interpreted with caution. Up to date, both CSF and plasma sTREM2 levels have been proposed as significant predictive biomarkers for progression from MCI to AD, with lower CSF sTREM2 levels indicating an elevated risk of disease progression (Zhou et al., 2023). The newly reported TREM2 p.W44X variant in one of our Peruvian families further supports the pathogenic relevance of heterozygous premature truncating *TREM2* mutations in AD, with some affected patients reported as having “typical” AD pathology (Guerreiro R. et al., 2013). Given the potential impact on sTREM2 levels and the role of TREM2 in neuroinflammation, these findings highlight the need to complement genetic findings with functional studies and underscore the importance of investigating underrepresented populations to fully understand the genetic architecture of AD. The phenotypic contrast between homozygous loss-of-function TREM2 mutations causing Nasu–Hakola disease (NHD/PLOSL) and heterozygous truncating or rare missense variants that increase the risk of AD and FTD suggests a dose-dependent effect of TREM2 dysfunction (Paloneva et al., 2002; Guerreiro R. et al., 2013; Jonsson et al., 2013; Borroni et al., 2014). In the homozygous state, near-complete loss of TREM2 function leads to a severe systemic and neurodegenerative phenotype (Kleinberger et al., 2014), whereas partial loss of function in heterozygous carriers may be sufficient to modulate microglial responses to amyloid and other insults, thereby increasing susceptibility to AD without producing the full NHD/PLOSL syndrome. In this context, heterozygous W44X in our family may act as a risk factor for relatively late-onset AD rather than a fully penetrant Mendelian cause.

Although the segregation of the variant within the family and the presence of similar pathogenic truncating mutations (e.g., Q33X, W78X, W191X) support a likely pathogenic role, these lines of evidence do not definitively establish causality. In the absence of biomarker confirmation and functional assays, misclassification or coincidental segregation cannot be entirely excluded. Therefore, the pathogenicity of TREM2 p.W44X should be considered provisional until functional and biomarker-supported studies are available. For TREM2 p.W44X, although the variant meets ACMG/AMP criteria for a Likely Pathogenic loss-of-function allele (Richards et al., 2015), heterozygous *TREM2* variants are not fully penetrant causes of autosomal-dominant Alzheimer’s disease. Rather, they are recognized as genetic risk modifiers that increase susceptibility to AD with incomplete penetrance (Jin et al., 2014; Jonsson et al., 2013; Yang et al., 2025; Guerreiro R. et al., 2013). This distinction is crucial, as the pathogenic effect of *TREM2* loss-of-function variants is recessive in Nasu–Hakola disease, whereas heterozygous carriers exhibit variable and probabilistic contributions to AD risk.

The second genetic variant reported in this study is a novel heterozygous variant in *PSEN1* (PSEN1 p.A431T; no rs(); Chr14:73685884 G > A), a gene highly associated with familial cases of AD. This mutation falls within exon 12 (out of 13 exons) and causes the change of an alanine for a threonine at position 431 of the protein. This mutation was found in the proband (II.1), an affected sibling (II.5) and other three asymptomatic relatives (II.2, III.3 and III.5).

Interestingly, two other similar mutations have been reported at the same amino acid position. One of them is PSEN1 p.A431E, known as the “Jalisco” mutation, which has been reported in several AD familial cases which descended from a common ancestor in Jalisco, Mexico (Orozco-Barajas et al., 2022). PSEN1 p.A431E affected individuals had an average age of onset of 42.5 years and a mean disease duration of 7.5 years, with substantial clinical heterogeneity in disease presentation (Dumois-Petersen et al., 2020). The second mutation at position A431 is PSEN1 p.A431V. It was found in a Japanese man with a family history of early onset dementia affecting six members, spanning three generations. In this case, the proband presented with mild cognitive impairment (MCI) at age 47 and converted to AD 16 months later (Matsushita et al., 2002). A biomarker study performed in the cerebrospinal fluid (CSF) of the proband, 1 day post-MCI diagnosis, showed normal A*β*42 levels but elevated tau and phosphorylated tau levels, which aligns with AD biomarker profiles.

The PSEN1 protein is organized into nine transmembrane helices (TM1–TM9), with its N-terminus facing the cytosol and its C-terminus oriented toward the lumen (Laudon et al., 2005). Mutations in the transmembrane domains (TM) of the PSEN1 protein—specifically TM1, TM2, TM3, TM4, and TM6—have been implicated in the pathogenesis of Alzheimer’s disease (AD; Hardy and Crook, 2001). The residue A431 is located within the cytosolic region, positioned in the middle of a short amino acid stretch that bridges TM8 and TM9 of PSEN1. Notably, this site lies near the highly conserved “PALP” motif (residues P433, A434, L435, and P436), situated near the N-terminal boundary of TM9. Mutations within this motif, particularly A436S, have also been directly associated with early-onset familial AD (Arber et al., 2024).

The precise biological effect of mutations A431E and A431V (and A431T by extension) remains unknown, however a cryo-electron microscopy study of the interaction of *γ*-secretase bound to an APP fragment suggests that the A431 residue interacts with the *β*-strand of APP, which is crucial for *γ*-secretase cleavage (Zhou et al., 2019). The occurrence of multiple pathogenic substitutions at the same amino acid in different populations highlights the importance of the amino acid position 431 in AD pathogenesis and the relevance of studying populations of non-European ancestry to confirm genetic associations with AD.

The clinical presentation of PSEN1 p.A431T in our family appears later and is potentially less penetrant than the well-known A431E (‘Jalisco’) variant, which typically presents during the early 40s (Meraz-Ríos et al., 2022; Murrell et al., 2006). Two A431T carriers in our family remain asymptomatic beyond age 40 (II.2 and III.3), suggesting a milder functional impact. Although both mutations occur adjacent to the highly conserved PALP motif and near the APP β-strand interaction site within γ-secretase, the biochemical nature of the substitutions differs substantially: A431E introduces a negatively charged, bulkier residue expected to disrupt γ-secretase–APP interactions more markedly, while A431T introduces a small polar residue that may alter local hydrogen bonding but is less structurally disruptive. This difference may partly account for the later clinical expression and possible reduced penetrance observed in A431T carriers.

In accordance with the pathogenicity framework proposed by Hsu et al. (2018), the PSEN1 p.A431T variant does not meet the criteria for classification as pathogenic or likely pathogenic. Segregation evidence is limited to two affected carriers, Moreover, the ages at diagnosis (65 and 73 years) are substantially later than the mean onset of ~45 years typically observed for pathogenic PSEN1 variants, while two unaffected carriers are also older than 45 years and remain assymtomatic. No functional studies are available. Although other substitutions at this residue (A431E, A431V) have been reported as pathogenic and the site lies adjacent to the PALP motif important for *γ*-secretase function, positional evidence alone is insufficient for pathogenic classification. Therefore, we propose that PSEN1 p.A431T be considered a Variant of Uncertain Significance (VUS) until more robust segregation, functional, and biomarker data become available.

In terms of study limitations, we report the following: First, AD-related variants were identified in only two of the 14 families screened, and the number of affected and unaffected carriers within each pedigree is small. This limited sample size precludes precise estimation of age-dependent penetrance, segregation patterns, and phenotypic expressivity for TREM2 p.W44X and PSEN1 p.A431T. As such, our observations should be viewed as preliminary and hypothesis-generating rather than definitive. Larger familial cohorts, longer longitudinal follow-up of currently unaffected carriers, and integration with regional consortia (such as ReDLat) will be required to more accurately quantify the risk associated with these variants and to delineate their clinical heterogeneity.

A second important limitation of our study is the absence of functional validation. For both TREM2 p.W44X and PSEN1 p.A431T, our classification as ‘likely pathogenic’ or VUS relies on segregation patterns, *in silico* pathogenicity prediction tools, and analogy to previously reported variants affecting the same domains or residues (e.g., TREM2 truncating mutations such as Q33X, and PSEN1 A431E/A431V). However, we did not perform experimental assays to measure protein expression, γ-secretase activity, ligand binding, or microglial signaling in TREM2, nor did we have access to CSF or plasma biomarkers such as sTREM2, Aβ42/40 ratio, p-tau, or neurofilament light.

Likewise, a third limitation was the lack of biomarkers as no blood/CSF nor amyloid or tau PET imaging was available. In Peru, cerebrospinal fluid (CSF) assays and amyloid/tau PET imaging are not part of the standard clinical practice, and structural MRI is inconsistently accessible (Custodio et al., 2022). Consequently, AD diagnoses were based on detailed neuropsychological assessments, clinical criteria and expert consensus; therefore, some degree of diagnostic uncertainty must be acknowledged—particularly for variants such as TREM2 p.W44X, which may increase risk for multiple neurodegenerative phenotypes rather than a single disease entity. Thus, while existing evidence suggests a plausible pathogenic role, these variants cannot be definitively classified as pathogenic without functional or biomarker-based confirmation. Future studies incorporating biochemical assays and biomarker-supported phenotyping will be essential to establish the biological consequences of these variants. Similarly, APOE genotyping was performed on probands but not their families. Although APOE ε4 is an important common risk factor for LOAD, the primary focus was on rare coding variants in neurodegeneration-related genes, and APOE status would not have substantially changed the interpretation of the TREM2 and PSEN1 variants reported here. Nonetheless, the lack of APOE data can be considered as an additional limitation, and future studies in larger Peruvian cohorts should consider integrating APOE and other polygenic risk markers to better contextualize individual and familial risk.

A fourth important limitation of our study, is the fact that the variant analysis focused on a predefined panel of genes previously associated with neurodegenerative diseases. The 119 genes included in our targeted analysis were selected because they encompass the major genetic pathways and mechanisms implicated in neurodegenerative diseases with cognitive manifestations. This curated panel integrates genes with established or suspected roles in Alzheimer’s disease, frontotemporal dementia, Lewy body disease, prion disorders, hereditary leukoencephalopathies, lysosomal and autophagy defects, microglial immune signaling, and synaptic dysfunction. The panel was assembled from multiple sources: (1) genes consistently included in clinically validated diagnostic panels for neurodegeneration; (2) genes with known pathogenic or likely pathogenic variants reported in OMIM, ClinVar, and peer-reviewed literature; and (3) genes with strong biological relevance to pathways central to AD pathophysiology, including amyloid processing (APP, PSEN1/2), innate immune and microglial function (TREM2, TYROBP, CSF1R), lipid metabolism (APOE, ABCA7, CLU), vesicle trafficking and endolysosomal function (CHMP2B, OPTN, PINK1), and tau biology (MAPT, MARK2, MARK4). The rationale for using a biologically informed, literature-curated panel—rather than agnostic genome-wide variant discovery—was to prioritize variants with the highest prior probability of mechanistic relevance given our small sample size and limited statistical power. This approach reduces false positives and enhances interpretability, particularly in resource-limited settings. However, we acknowledge that because many known neurodegeneration genes have been identified in predominantly European-ancestry cohorts, this strategy may underdetect population-specific or novel variants in Peruvian individuals. We explicitly recognize this as a limitation of our study and emphasize that future work employing exome-wide or genome-wide approaches, ideally in larger and ancestrally diverse cohorts, will be needed to uncover the full spectrum of genetic risk in Latin American populations.

Taken together, the identification of TREM2 (p.W44X) and PSEN1 (p.A431T) variants in Peruvian families with familial Alzheimer’s disease (AD) carries relevant scientific, clinical, and public health implications. Scientifically, these findings expand the genetic architecture of AD in underrepresented Latin American populations, addressing a critical gap in global dementia research, as well as other health conditions (Sirugo et al., 2019). Previous studies by our research group have shown that the Peruvian population have a South American autochthonous genetic background of about 70% (Sandoval et al., 2013). The identification of a *TREM2* truncating variant in a familial case of AD reinforces the role of microglial dysfunction in AD pathogenesis, supporting emerging immunomodulatory therapies, while the novel *PSEN1* mutation broadens the spectrum of causal variants for early-onset AD, with potential implications for amyloid-targeted treatments. Clinically, this work highlights the urgent need for accessible genetic testing and counseling in low-resource settings, enabling early diagnosis and personalized risk management for at-risk families. From a public health perspective, these results underscore the necessity of integrating genetic screening into national dementia strategies in Latin America, where aging populations face a looming epidemic of AD. By linking these discoveries to innovative approaches—such as biomarker-guided prevention trials and telehealth-based family monitoring—our study provides a framework for precision medicine in diverse and underserved populations, ultimately advancing equitable care and therapeutic development for Alzheimer’s disease worldwide. In conclusion, this study identifies two rare genetic variants in *TREM2* and *PSEN1* in Peruvian families with AD and highlights the need for further investigation of these alleles in larger cohorts. While our results contribute to expanding the genetic data available from underrepresented populations, the limited segregation information, absence of biomarker and functional data, and small sample size prevent definitive conclusions regarding pathogenicity or clinical actionability. Future work integrating broader genomic analyses, functional studies, and regionally representative datasets (including collaborations with Latin American consortia such as ReDLat) will be essential to clarify the penetrance, expressivity, and biological impact of these variants. Finally, our findings underscore the importance of expanding genomic research in diverse populations.

## 5 Conclusion

This study describes 02 novel genetic associations with AD in two Peruvian families. The first, a heterozygous variant in *TREM2* (p.W44X) was found in the proband (71yo at clinical diagnosis), his two affected siblings (73 and 80yo at clinical diagnosis) and a third asymptomatic mutation carrier relative of 57yo at the time this study was published. Although this truncating mutation has already been associated with NHD/PLOSL when in homozygosity, its precise biological effect in AD remains to be elucidated by future studies.

The second mutation was a novel heterozygous variant in *PSEN1* (A431T). The proband and one sibling were affected carriers of the mutation. Age at clinical diagnosis for the proband was 65yo, while his sister was diagnosed at 73. A third sibling and other two family members were also carriers of the variant, but younger than 60yo when recruited. This is the first report of *PSEN1* A431T associated with AD. However, based on current evidence and following Hsu et al. criteria, this variant should be considered a VUS rather than definitively pathogenic or likely pathogenic. Remarkably, other pathogenic and likely pathogenic mutations have already been reported at the same position (A431E and A431V). Further *in vitro* functional analyses are needed to better elucidate the role of the A431 mutations in AD.

## Data Availability

WES data for both probands (BioProject ID PRJNA1411046) can be found in the following repository link: http://www.ncbi.nlm.nih.gov/bioproject/1411046.
